# Cytokine imprinting - mechanisms for memory

**DOI:** 10.1186/ar3416

**Published:** 2011-09-16

**Authors:** Andreas Radbruch

**Affiliations:** 1 Deutsches Rheuma-Forschungszentrum Berlin, ein Leibniz Institut, Charitéplatz 1, 10117 Berlin, Germany

## 

Imprinting of T helper lymphocytes for the reexpression of cytokines is crucial for protection against recurring pathogens but also can be a driving force of chronic inflammation. Th1 and Th17 cells are distinct lineages of proinflammatory effector/memory cells, imprinted for reexpression of interferon-γ (IFN-γ) and interleukin-17 (IL-17), by upregulated expression of the transcription factors T-bet and RORγt, respectively. Imprinting here means that while expression of the cytokine genes upon primary instruction requires signals from both, the T cell receptor and receptors for instructing cytokines, reexpression requires only T cell receptor signaling in reactivated effector/memory T cells. Interleukin-12 (IL-12) and IFN-γ are essential instruction signals for the differentiation of Th1 cells and the imprinting of the *Ifng *gene. In activated naïve T cells, IFN-γ induces the central Th1 transcription factor T-bet, while T-bet induces the expression of IFN-γ, in a positive, T cell receptor dependent feedback loop polarizing the T cell into Th1 differentiation. At this time, expression of the IL-12 receptor β2 chain (IL12Rβ2) is repressed by T cell receptor signaling. After TCR signaling has ceased, i.e. the antigen is eliminated, the IL12Rβ2 chain is expressed and IL-12 triggers a second wave of T-bet expression. This "late" T-bet expression coincides with expression of the transcription factors Hlx and Runx3, and it is required for imprinting of Th1 cells for the reexpression of IFN-γ. The signals required for Th17 differentiation and imprinting are less clearly defined. While signals such as TGF-β and IL-6 lead to the differentiation of IL-17 expressing cell in vitro, such cells fail to reexpress IL-17 in the absence of these instructive signals. In contrast, *in vivo *generated Th17 cells, have a stable memory for reexpression of IL-17 in vitro, upon restimulation by antigen. Such cells are refractory to Th1 or Th2 polarizing signals. Th cells coexpressing IFN-γ and IL-17 have been observed *in vivo*. *Ex vivo *isolated Th17 cells can be converted into Th1+17 cells by combined IFN-γ and IL-12 signaling. IFN-γ is required to upregulate expression of the IL12Rβ2 chain, and IL-12 for Th1 differentiation. These Th1+17 cells stably coexpress RORγt and T-bet on the single cell level, and are imprinted for reexpression of both IFN-γ and IL-17. Thus, for T lymphocytes, polarization and imprinting of inflammatory responses is regulated by dynamic interaction of IFN-γ and IL-12, and regulation of expression of the IL-12 receptor.

